# Novel *M tuberculosis* Antigen-Specific T-Cells Are Early Markers of Infection and Disease Progression

**DOI:** 10.1371/journal.pone.0028754

**Published:** 2011-12-28

**Authors:** Davinder P. S. Dosanjh, Mustafa Bakir, Kerry A. Millington, Ahmet Soysal, Yasemin Aslan, Serpil Efee, Jonathan J. Deeks, Ajit Lalvani

**Affiliations:** 1 Tuberculosis Research Unit, Department of Respiratory Medicine, National Heart and Lung Institute, Imperial College London, London, United Kingdom; 2 Department of Paediatrics, Marmara University School of Medicine, Istanbul, Turkey; 3 Unit of Public Health, Epidemiology and Biostatistics, School of Population Health and Sciences, University of Birmingham, Edgbaston, Birmingham, United Kingdom; Statens Serum Institute, Denmark

## Abstract

**Background:**

*Mycobacterium tuberculosis* Region-of-Difference-1 gene products present opportunities for specific diagnosis of *M. tuberculosis* infection, yet immune responses to only two gene-products, Early Secretory Antigenic Target-6 (ESAT-6) and Culture Filtrate Protein-10 (CFP-10), have been comprehensively investigated.

**Methods:**

T-cell responses to Rv3873, Rv3878 and Rv3879c were quantified by IFN-γ-enzyme-linked-immunospot (ELISpot) in 846 children with recent household tuberculosis exposure and correlated with kinetics of tuberculin skin test (TST) and ESAT-6/CFP-10-ELISpot conversion over six months and clinical outcome over two years.

**Results:**

Responses to Rv3873, Rv3878, and Rv3879c were present in 20–25% of contacts at enrolment. Rv3873 and Rv3879c responses were associated with and preceded TST conversion (P = 0.02 and P = 0.04 respectively), identifying these antigens as early targets of cell-mediated immunity following *M. tuberculosis* exposure. Responses to Rv3873 were additionally associated with subsequent ESAT-6/CFP-10-ELISpot conversion (P = 0.04). Responses to Rv3873 and Rv3878 predicted progression to active disease (adjusted incidence rate ratio [95% CI] 3.06 [1.05,8.95; P = 0.04], and 3.32 [1.14,9.71; P = 0.03], respectively). Presence of a BCG-vaccination scar was associated with a 67% (P = 0.03) relative risk reduction for progression to active tuberculosis.

**Conclusions:**

These RD1-derived antigens are early targets of cellular immunity following tuberculosis exposure and T-cells specific for these antigens predict progression to active tuberculosis suggesting diagnostic and prognostic utility.

## Introduction

Eradication of tuberculosis in low- and intermediate-burden regions hinges on the accurate diagnosis and treatment of latent tuberculosis infection (LTBI) [1], [2]. The advent of interferon-γ release assays (IGRAs) is a significant advance in the diagnosis of *M. tuberculosis* infection [3], [4]. The chief advantage of IGRAs over the tuberculin skin test (TST) is their high specificity for *M. tuberculosis* infection [5], [6], [7], [8], [9], [10], [11] which stems from the genomic origins of the antigens they use: early secretory antigenic target-6 (ESAT-6) and culture filtrate protein-10 (CFP-10). Both gene products are encoded in Region of Difference-1 (RD1), a genomic segment present in *M. tuberculosis* complex but absent from all strains of BCG and most environmental mycobacteria [12], [13], [14], [15].

T-cell responses to these antigens are prognostic of progression to active tuberculosis disease but from the few longitudinal cohort studies published to date there is no clear consensus as to whether the prognostic power of current ESAT-6/CFP-10-based IGRAs is significantly greater than the TST [16], [17], [18], [19], [20], [21], [22], [23], [24]. Development of a biomarker with higher predictive power than TST for progression to active tuberculosis is a global public health priority as it would greatly reduce the number of contacts that need to be treated to prevent each case of active disease [25], [26], [27], [28]. Progress towards such a biomarker requires investigation of immune responses to a broad range of candidate *M. tuberculosis* antigens in tuberculosis contacts and correlation with clinical outcomes prospectively over time; however, no longitudinal studies have investigated antigens other than those in the commercial IGRAs: ESAT-6, CFP-10 and, in the case of Quantiferon-Gold-In tube, Rv2654.

The remaining RD1-encoded gene products share the same species distribution as ESAT-6 and CFP-10, are highly specific for *M. tuberculosis* complex but have been little-investigated to date [29], [30], [31], [32], [33], [34], [35], [36], [37]. The limited available data derived from cross-sectional studies in active tuberculosis disease and BCG-vaccinated unexposed controls indicate that interferon-γ (IFN-γ) T-cell responses to these antigens have high specificity (97.4%–100%) and low-to-moderate sensitivity (25.5%–53.1%) for active tuberculosis, after exclusion of cross-reactive peptides encoded in conserved motifs of Rv3873 [30], [35], [37]. Given their location in the *M. tuberculosis* genome and their high specificity in tuberculosis-unexposed controls, responses to these antigens in LTBI are of considerable interest. Whether they are targeted by early T-cell responses after recent tuberculosis exposure and whether these responses correlate with clinical outcomes over time are important questions for advancing our understanding of how *M. tuberculosis*-specific cellular immunity evolves after exposure and for development of better biomarkers of LTBI.

We therefore investigated T-cell responses to Rv3873, Rv3878 and Rv3879c after tuberculosis exposure and correlated responses with progression to active tuberculosis disease and established markers of recent infection, i.e. TST conversion and ESAT-6/CFP-10-ELISpot conversion, in a well-characterized cohort of children with a very low prevalence of HIV infection and recent household tuberculosis exposure [17], [38], [39].

## Materials and Methods

### Ethics Statement

Four-hundred forty-three adults with sputum smear-positive pulmonary tuberculosis in the Istanbul government tuberculosis service during the 20-month enrolment period with ≥1 child household contact gave written informed consent on behalf of the children and 1,024 child contacts aged ≤16 years were enrolled at Marmara University Hospital [38].

Ethical approval was granted by the Institutional Review Board of Marmara University Hospital, The Turkish Ministry of Health and the World Health Organization Steering Committee on Research Involving Human Subjects.

### Clinical evaluation and follow-up

All children had a medical history, physical examination, documentation of BCG-vaccination scars, TST and chest-radiography and 1,020 gave a 10 mL blood sample. Initial venesection for ELISpot and inoculation of PPD for TST were performed on the same day for 1,007 of these children during the 20-month period beginning October 2002. All assays were performed during this period. In accordance with national guidelines, all children are BCG-vaccinated aged two-three months and again at six-seven years old. BCG coverage in children in Turkey was 79% in 2004 [38], [40]. No children underwent repeat BCG vaccination during the course of the study. All contacts were invited for clinical follow-up six-monthly for two years, and parents advised to return with the child for assessment if intercurrent symptoms developed.

Six-months isoniazid preventive therapy (IPT) was given in accordance with national guidelines [41] to children <6-years old; children ≥6-years old with a positive TST or converted second TST. Parents or guardians, who were provided with tablets at 2 monthly intervals, administered isoniazid to their children. We questioned all parents or guardians who returned for follow-up about adherence to preventive treatment, and all reported full adherence [17].

### Case definitions

Study pediatricians, blinded to the ELISpot results, diagnosed incident tuberculosis on the basis of clinical, radiologic, and microbiological criteria (see Materials and Methods S1) [17].

### Tuberculin skin test

TST using the Mantoux method and two tuberculin units purified protein derivative (PPD) RT23 was administered and read by study pediatricians (blinded to ELISpot results) (see Materials and Methods S1). For our analysis, TSTs were scored positive where induration was ≥5 mm [17], [42]. TST-negative contacts had a repeat TST two-six months later to identify TST conversion, defined as an increase of ≥10 mm [42].

### ELISpot assays

These were performed and read by persons blind to personal identifiers and TST results as described [38]. Pairs of duplicate wells (2·5×10^5^ peripheral blood mononuclear cells [PBMCs] per well) contained: positive (mitogen) and negative (no stimulus) controls, streptokinase-streptodornase, PPD and 1 of 14 peptide-pools incorporating 15-mer peptides spanning the length of ESAT-6 (n = 17), CFP-10 (n = 18), or selected sequences [30] from Rv3873 (n = 12), Rv3878 (n = 14) and Rv3879c (n = 17). Previously-described non-specific peptides from Rv3873 were excluded [30]. The ESAT-6/CFP10-peptide assay was subsequently commercialized into the regulatory-approved T-SPOT.*TB* (Oxford Immunotec, Oxford, United Kingdom). Wells were scored as previously described [38]. ELISpot assays were repeated six months post-recruitment (Materials and Methods S1). Repeat assays prioritized the use of the conventional antigens and hence due to lack of PBMCs only ESAT-6/CFP-10 peptide pools were included.

### Statistical analysis

We defined each child's entry into the study as the date first examined and tested with ELISpot and TST; the end-point was development of active tuberculosis or the last follow-up assessment (whether by telephone or clinic visit). All analysis was based on results obtained from assays done at the time of the original study. Differences between proportions of contacts responding to each antigen were compared using McNemar's test for paired binary variables. The magnitude of antigen-specific T-cell responses amongst responders was compared using Wilcoxon matched-pairs test for continuous variables. We used Poisson regression to estimate incidence rates of progression to tuberculosis per 1000 person-years follow-up, with 95% confidence intervals (CIs). We estimated incidence rate ratios to compare the prognostic value of test-positive with test-negative results, both unadjusted and adjusted for IPT. The relative risk (RR), with 95% CIs, was calculated for TST and ELISpot conversion. We did all analyses in Stata, v9.1 (StataCorp, College Station, Texas).

## Results

### Demographic and clinical characteristics of participants

**Figure 1:**
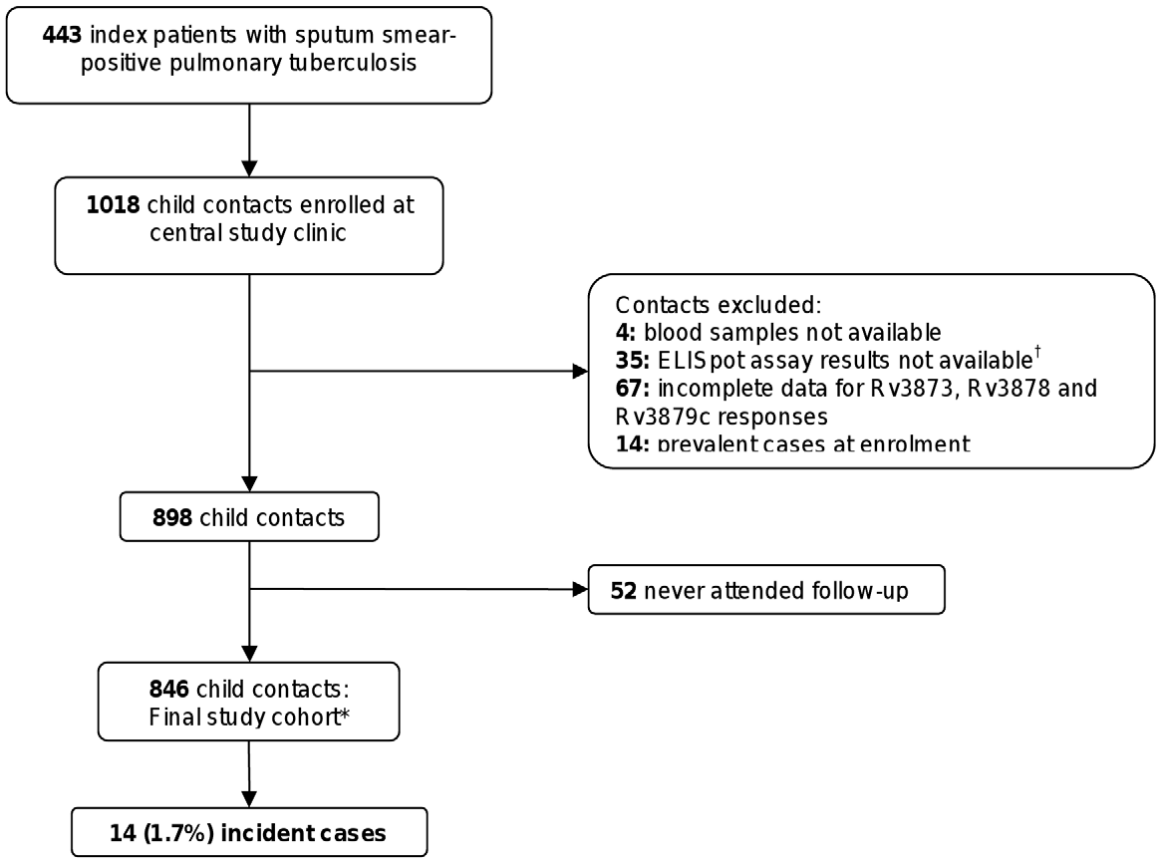
Study flow diagram. *The final study cohort of 846 child contacts with complete data on responses to all tested antigens was followed for 1125 person-years (mean duration: 1.04 years follow-up [range 0.25 to 3.28 years]). Fifty-seven children were lost to further follow-up after clinical assessment at six months; 256 children completed 24 months follow-up with six-monthly clinical reviews and 519 children were followed every six months by standardized health status telephone questionnaire administered to parents and guardians by the study nurse after the first 12 months of six-monthly clinical reviews. ^†^Two contacts were removed due to loss of the ELISpot plates and thirty-three contacts were removed due to an episode of bacterial contamination of peptide pool reagents [38].

Of 1024 children enrolled, 846 were confirmed household contacts of adults with sputum smear-positive pulmonary tuberculosis with complete demographic, TST and ELISpot data for all antigens available at recruitment (Figure 1). These 846 children with a median age of 90 months (IQR 48 to 132), were contacts of 401 index patients living in 382 separate households, with 18 households having more than one adult index patient with smear-positive pulmonary tuberculosis. 50.5% were male, and the average number of contacts per household was 2.4. Six-hundred seventy-nine contacts (80%) had a BCG scar.

The study cohort of 846 child contacts was followed for 1125 person-years with a mean duration of 1.04 years follow-up (range:0.25 to 3.28 years). Six-hundred and five children received IPT. Fifty-seven children were lost to further follow-up after clinical assessment at six months; withdrawal rates did not differ according to ESAT-6/CFP-10 ELISpot status (6% of contacts with positive ELISpot results vs 7% with negative results).

Fourteen contacts (1.7%) developed active tuberculosis disease during the follow-up period. Sixty-four percent of contacts who progressed to active disease were female, with a median age of 49 months (range:4 to 177) [17]. Eight (57%) had BCG scars compared with 671 of the 832 (81%) (P = 0.03, Chi-square test) who did not progress to tuberculosis (RR = 67% (95% CI: 7%–88%). All 14 incident cases were asymptomatic with normal chest radiography at enrolment and had no past history of active tuberculosis. Of the 12 incident cases started on IPT at enrolment, six developed active tuberculosis before completing the course and three were exposed to index cases with multi-drug resistant tuberculosis [17]. Twelve patients developed pulmonary tuberculosis and two developed miliary disease [17]. The median time from recruitment to progression to active disease was six months (range:4 to 25). Although only three of 14 cases were culture-confirmed, all had definitive clinical and radiological diagnoses, an appropriate response to anti-tuberculosis treatment and remained well for >1 year after treatment completion [17].

### Hierarchy of immunodominance of the RD1-encoded antigens

**Figure 2:**
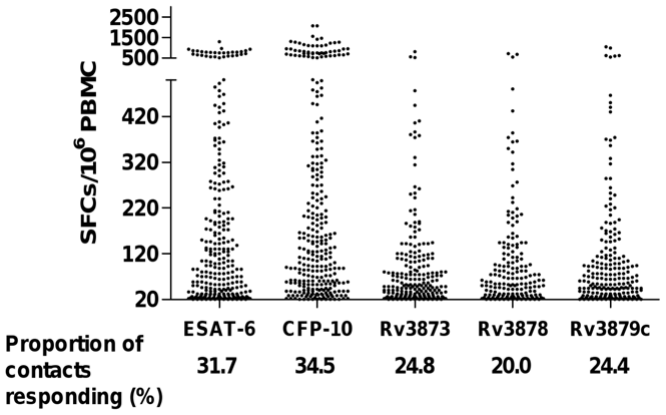
Magnitude of IFN-γ T-cell responses to each RD1-encoded antigen among responders to each antigen. The median (inter-quartile range) of antigen-specific T-cells in individuals who had a positive ELISpot response to ESAT-6, CFP-10, Rv3873, Rv3878 and Rv3879c were: 104 (38, 274.5), 133 (55.5, 348.5), 52 (30.5, 110), 62 (30, 124) and 64 (32, 115.5) per million PBMCs respectively. Significantly more contacts had T-cell responses to CFP-10 (P<0.001) and ESAT-6 (P<0.002) than to any of the novel antigens. Fewer contacts mounted a T-cell response to Rv3878 than any other antigen (P<0.001 in all cases) and the proportion of contacts with T-cell responses to Rv3873 and Rv3879c did not significantly differ from each other. CFP-10 responses were significantly greater than responses to any other antigen (P<0.02 in all cases). The peptide-specific IFN-γ-secreting T-cell response specific for ESAT-6 was significantly greater compared to the RD1-derived novel antigens (P<0.001 in all cases).

The T-cell responses to Rv3873, Rv3878, Rv3879c, ESAT-6 and CFP-10 for each contact are presented in Figure 2. Significantly more contacts had responses to CFP-10 (P<0.001) and ESAT-6 (P<0.002) than to any of the novel antigens. Fewer contacts mounted a T-cell response to Rv3878 than any other antigen (P<0.001 in all cases) and the proportion of contacts with T-cell responses to Rv3873 and Rv3879c did not significantly differ from each other.

Comparing the number of IFN-γ-spot forming cells for each antigen, CFP-10 responses were significantly greater than responses to any other antigen (P<0.02 in all cases). The T-cell response specific for ESAT-6 was significantly greater compared to the novel antigens (P<0.001 in all cases).

### T-cell responses to RD1-encoded antigens after recent tuberculosis exposure precede and predict TST conversion

Three-hundred thirty-one (39.1%) child household contacts exhibited a negative TST result at the time of recruitment. Three-hundred ten (94%) initially TST-negative contacts had a repeat TST two to six months later (237 [76%] of which were done within four months) and 41 (13%) of these became TST-positive. TST-negative contacts with a T cell response to Rv3873, Rv3879c or PPD at recruitment were significantly more likely to become TST-positive at the time of the second TST (Table 1).

**Table 1:** Relative risk of TST conversion for ELISpot-positive, TST-negative contacts.

	ESAT-6		CFP-10		Rv3873		Rv3878		Rv3879c		PPD		SKSD^a^	
Test result	positive	negative	positive	negative	positive	negative	positive	negative	positive	negative	positive	negative	positive	negative
**TST conversion** b	3	38	4	37	7	34	3	38	8	33	26	15	21	20
**TST non-conversion**	25	244	19	250	17	252	10	259	24	245	114	155	159	110
**Cumulative percentage risk of conversion (95% CI)**	10.7	13.5	17.4	12.9	29.2	11.9	23.1	12.8	25	11.9	18.6	8.8	11.7	15.4
	(2.2, 28.2)	(9.7, 18.0)	(5.0, 38.8)	(9.2, 17.3)	(12.6, 51.1)	(8.4, 16.2)	(5.0, 53.8)	(9.2, 17.1)	(11.5, 43.4)	(8.3, 16.3)	(12.5, 26.0)	(5.0, 14.1)	(7.4, 17.3)	(9.6, 22.8)
**Relative risk of conversion (95% CI)**	0.80 (0.26, 2.41)		1.35 (0.53, 3.45)		2.45 (1.22, 4.93)		1.80 (0.64, 5.08)		2.11 (1.07, 4.16)		2.10 (1.16, 3.82)		0.76 (0.43, 1.34)	
**P value**	0.68		0.54		0.02		0.28		0.04		0.01		0.34	

a Streptokinase streptodornase (SKSD), a non-tuberculosis control antigen.

b TST conversion defined as an increase in induration of ≥10 mm between the 1st and 2nd TST where the first TST was <5 mm.

### T-cell responses to Rv3873 after recent tuberculosis exposure precede and predict ESAT-6/CFP-10 specific IFN-γ ELISpot conversion

Four-hundred eighty-six (57%) contacts had no detectable IFN-γ T-cell response to ESAT-6/CFP-10 at recruitment. Three-hundred eighty seven (80%) of these initially ESAT-6/CFP-10-negative contacts had a repeat ELISpot carried out five to seven months post-recruitment, by which time-point 59 (15%) exhibited an ESAT-6/CFP-10-specific T-cell response.

Contacts with no baseline response to ESAT-6/CFP-10 who had T-cell responses to Rv3873 (n = 32) were significantly more likely to develop an ESAT-6/CFP-10-specific T-cell response by the time of the second ELISpot (relative risk of conversion 2.22 (95% CI 1.03, 4.81) P = 0.04), suggesting that Rv3873 is an earlier target of *M. tuberculosis*-specific T-cells after *M. tuberculosis* exposure than ESAT-6/CFP-10. Baseline T-cell responses to Rv3878, Rv3879c, PPD and SKSD as well as TST results were not significantly associated with development of ESAT-6/CFP-10 T-cell responses six months later (data not shown).

### Baseline responses to the novel RD1-encoded antigens detect potentially infected contacts who did not respond to ESAT-6/CFP-10

After taking ESAT-6/CFP-10 responses into account, responses to Rv3873, Rv3878 and Rv3879c identified 54 (6.4%) further contacts as infected with *M. tuberculosis* at recruitment (P<0.001, McNemar's test). With ESAT-6/CFP-10 and the novel antigens combined, 414 (48.9%) contacts were identified as being infected with *M. tuberculosis*.

### T-cell responses to Rv3873 and Rv3878 after M. tuberculosis exposure predict progression to active disease

Of 210 contacts with positive IFN-γ T-cell responses to Rv3873 at recruitment, seven (3.3%) progressed to active tuberculosis and of 169 contacts with responses to Rv3878, six (3.6%) progressed. Responses to each of these individual antigens predicted progression to active tuberculosis on unadjusted and adjusted analyses (Table 2). Responses to either ESAT-6 or CFP-10 each detected eight incident cases, but from a larger group of positive responders. Predictive values of responses to ESAT-6 or CFP-10 were of borderline statistical significance at the 5% level on adjusted analysis only (Table 2). T-cell responses to PPD and SKSD at recruitment were not significantly associated with progression to active disease (Table 2). Although responses to Rv3873 identified a similar number of incident cases to ESAT-6 and CFP-10, a significantly lower proportion of contacts had positive responses to Rv3873 (210/846) than to ESAT-6 (272/846; p = 0.013) or CFP-10 (296/846; p = 0.001).

**Table 2:** Incidence rates of tuberculosis and incidence rate ratios among child contacts, by individual antigen ELISpot results at recruitment.

	ESAT-6		CFP-10		Rv3873		Rv3878		Rv3879c		PPD		SKSD^a^	
Test result	positive	negative	positive	negative	positive	negative	positive	negative	positive	negative	positive	negative	positive	negative
**Total results, n**	272	574	296	550	210	636	169	677	206	640	619	227	547	299
**Incident TB cases, n**	8	6	8	6	7	7	6	8	5	9	11	3	10	4
**Person years at risk**	384	740	411	714	294	831	225	899	290	834	826	298	710	414
**Unadjusted analysis**														
**Incidence rate per 1000 person years (95% CI)**	20.8 (9.0, 41.0)	8.1(3.0, 17.6)	19.4 (8.4, 38.3)	8.4(3.1, 18.2)	23.8 (9.6, 49.1)	8.4(3.4, 17.4)	26.6 (9.8, 58.0)	8.9(3.8, 17.5)	17.2 (5.6 40.1)	10.8 (4.9, 20.5)	13.3 (6.6, 23.8)	10.0 (2.1, 29.3)	14.1 (6.8, 25.9)	9.7(2.6, 24.7)
**Incidence rate ratio (95% CI)**	2.57 (0.89, 7.40)		2.32 (0.80, 6.68)		2.83 (0.99, 8.07)		3.00 (1.04, 8.63)		1.60 (0.53, 4.76)		1.32 (0.37, 4.74)		1.46 (0.46, 4.65)	
**P value**	0.08		0.12		0.05		0.04		0.4		0.67		0.52	
**Adjusted analysis** b														
**Incidence rate ratio (95% CI)**	2.97 (1.01, 8.73)		2.92 (0.99, 8.59)		3.06 (1.05, 8.95)		3.32 (1.14, 9.71)		1.69 (0.55, 5.17)		2.06 (0.55, 7.68)		3.26 (0.91, 11.70)	
**P value**	0.049		0.052		0.04		0.03		0.36		0.28		0.91	

a Streptokinase streptodornase (SKSD), a non-tuberculosis control antigen.

b Adjusted for age, sex, isoniazid preventive therapy given to contacts exposed to drug sensitive tuberculosis and BCG vaccination.

Ten incident cases had a positive response to ESAT-6/CFP-10 at recruitment; seven also responded to Rv3873, six to Rv3878 and five to Rv3879c. None of the incident cases had a positive response to Rv3873, Rv3878 or Rv3879c that did not also have a response to ESAT-6/CFP-10. Of the 360 contacts with a positive ELISpot response to ESAT-6/CFP-10, 10 progressed to active tuberculosis within 511 person years of follow-up (incidence rate, 19.6 per 1000 person years [95% CI, 9.3 to 36.0 per 1000 person-years]). Three hundred and thirty-four (93%) of these children, which includes all 10 incident cases, received IPT. On adjusted analysis, children with a positive ESAT-6/CFP-10 ELISpot result were 3.91 times more likely than those with a negative result to develop active disease (95% CI 1.20, 12.74, *P* = 0.02).

## Discussion

This cohort of children with household exposure to infectious pulmonary tuberculosis enabled, for the first time, investigation of T-cell responses to novel RD1-encoded antigens in a population of recently exposed individuals. Our findings identify these antigens as early targets of cellular immunity following exposure to *M. tuberculosis*. IFN-γ-secreting T-cells specific for these antigens had faster kinetics than established markers of recent *M. tuberculosis* infection and predicted progression to active tuberculosis.

With no gold standard test for LTBI, progression to active disease is the only way to confirm *M. tuberculosis* infection and is essential for defining the clinical utility of new biomarkers of LTBI. Baseline T-cell responses specific for Rv3873 and Rv3878 significantly predicted progression to active tuberculosis, indicating that the T-cell responses to these novel antigens are not only specific, but also prognostic of *M. tuberculosis* infection. These are the first antigens, other than those used in the commercial IGRAs, to be identified as having prognostic value. Moreover, only one other study has dissected the prognostic power of T-cell responses to the individual antigens contained in IGRAs: Leung et al found that IFN-γ-ELISpot responses to CFP-10 and not ESAT-6 were predictive of progression to tuberculosis in silicotic adults without recent tuberculosis contact [21]. In our study, the prognostic power of ELISpot responses to CFP-10, ESAT-6, Rv3873 and Rv3878 were broadly similar on multivariate analysis. Interestingly, responses to Rv3873 were significantly more specific for progression to active tuberculosis compared to responses to ESAT-6 or CFP-10. However, given the relatively low sensitivity of the T-cell response to these antigens in active disease, these antigens could not replace ESAT-6/CFP10 in IGRAs [30], [37].

The novel antigens did not identify any incident cases in addition to those already detected by ESAT-6/CFP-10 responses. Since 10 of the 14 contacts which progressed to active disease were identified by responses to ESAT-6/CFP-10, demonstration of incremental prognostic power would have necessitated the presence of responses to the novel antigens in one or more of the four ESAT-6/CFP-10 ELISpot-negative contacts who progressed to tuberculosis. Hence prospective studies with a greater number of ESAT-6/CFP-10-negative incident cases, as already observed in larger studies [22], [43], are required to fully evaluate the potential incremental value of these novel antigens.

Although predictive values are sometimes reported in studies of prognosis, their value is limited as they change with length of follow-up (positive predictive values increasing and negative predictive values decreasing as more events occur). The data needed to compute these values is presented in table two but we caution against their over-interpretation.

It must be noted that all children that were <6 years old, and children ≥6 years old that exhibited a positive TST result at recruitment, or subsequently converted their TST, were given a 6-month course of IPT in accordance with Turkish Ministry of Health guidelines [41]. Six-months isoniazid has only 60% efficacy which likely accounts for the substantial residual rate of progression to tuberculosis observed in this cohort [17], [44]. Since all TST-positive individuals received IPT the prognostic value of the TST could not be fairly assessed in this cohort [17]. Children who had T-cell responses to the *M. tuberculosis*-derived antigens were also more likely to have received IPT because of their significant association with positive TST results [17], [45]. Our results are therefore, if anything, an underestimate of the prognostic power of specific T-cell responses to these deleted region antigens. Given the ethical imperative for prompt IPT in young children exposed to tuberculosis, it may not be possible to robustly and definitively assess the true prognostic value of any test in the absence of TST-guided preventive treatment [17].

TST conversion is a long-established reference standard for recent *M. tuberculosis* infection. However, little is known about the comparative kinetics of development of cutaneous delayed-type hypersensitivity responses to tuberculin and circulating T-cells specific for defined *M. tuberculosis* antigens early after exposure to tuberculosis [43]. Our study illustrates the ability of *ex-vivo* IFN-γ T-cell responses to two of the novel antigens, Rv3873 and Rv3879c, as well as PPD, to predict TST conversion. The fact that T-cell responses to the novel antigens are associated with subsequent development of a cutaneous response to tuberculin is further evidence that they signal recent *M. tuberculosis* infection.

The development of IFN-γ-secreting T-cell responses to ESAT-6/CFP-10 after recent exposure is increasingly regarded as evidence of *M. tuberculosis* infection, and so the relationship between T-cell responses to the novel antigens and ESAT-6/CFP-10-ELISpot conversion was assessed. Rv3873-specific IFN-γ responses at recruitment in ESAT-6/CFP-10-ELISpot-negative contacts predicted subsequent development of ESAT-6/CFP-10-specific responses. These data further support the role of Rv3873 as a specific marker of *M. tuberculosis* infection and also indicate that this antigen induces an ultra-early T-cell response, preceding responses to ESAT-6 and CFP-10 which were hitherto considered to be the earliest antigenic targets following *M. tuberculosis* infection [46], [47], [48], [49].

Repeat TSTs and ELISpot assays were carried out between two and six, and five and seven months post-recruitment respectively. It is possible that some contacts were exposed to an additional index case between initial and subsequent testing. This is unlikely however, as the initial index cases were promptly treated, and contact tracing immediately carried out to identify and treat all potentially infected contacts [17], [38]. In addition, exposure risk outside the household was minimal compared to that within the household [38].

Given that T-cell responses to Rv3879c are an important marker of infection in active tuberculosis [30], [37], it is interesting to note that baseline Rv3879c-specific T-cell responses were not significantly associated with progression to active disease, although they were significantly associated with TST conversion. Conversely, responses to Rv3878 were predictive of progression to active tuberculosis but not of TST conversion. The reasons for these apparent anomalies are unclear however, it is the correlation of responses with disease progression that is the gold standard not correlation with TST conversion which is itself a limited and relatively insensitive marker for progression to TB.

Interestingly, presence of a BCG scar was associated with a 67% risk-reduction in progression to tuberculosis, consistent with its 50% protective effect in randomized trials [50]. This child cohort has thus demonstrated the protective effect of BCG on both risk-of-infection given exposure [38] and progression-to-disease given infection.

We have identified T-cell responses to selected sequences from Rv3873 and Rv3879c as early markers of *M. tuberculosis* infection and responses to Rv3873 and Rv3878 as prognostic markers of progression to tuberculosis in recently-exposed contacts. These data suggest that the 6.4% of child contacts who were negative at baseline to ESAT-6/CFP-10 but positive to one or more of these three antigens may have been infected with *M. tuberculosis*. However, even when including responses to the new antigens, only a small minority (<5%) of children with positive responses progressed to active TB, similar to the small proportion of responders who progressed in other longitudinal studies of IGRA in TB contacts and not substantially different to what has been historically observed with TST. The size of the baseline interferon-γ response did not significantly differ between incident cases and contacts that did not develop tuberculosis, suggesting that although a positive ELISpot result is prognostic of progression to tuberculosis, the magnitude of this response can not be used to further refine the risk for progression [17].

Quantification of the clinical utility of these new immunological markers will in practice depend on their incremental prognostic power when used in combination with the conventional IGRA antigens as well as their independent prognostic power when used alone. Future larger prospective studies that incorporate these novel antigens alongside ESAT-6 and CFP-10 will help to formulate the most predictive combination of antigens to meet the public health imperative to develop markers of LTBI with higher predictive power than the TST and existing IGRAs.

## Supporting Information

Materials and Methods S1 (DOC)
