# Supplementary material for: Novel M tuberculosis Antigen-Specific T-Cells Are Early Markers of Infection and Disease Progression
Source: PLoS One. 2011 Dec 28;6(12):e28754. doi: 10.1371/journal.pone.0028754 (PMC3247216; doi:10.1371/journal.pone.0028754)
Supplement: Materials and Methods S1 — (DOC) [file pone.0028754.s001.doc]

**Materials and Methods S1**

***Case definitions***

Two independent clinicians separately assessed the clinical and radiologic evidence for culture-negative incident cases, and in each case, the diagnosis was further confirmed by a documented successful clinical and radiologic response to antituberculosis treatment. We treated children with a diagnosis of tuberculosis with 6 months of standard chemotherapy, and we extended the continuation phase of isoniazid and rifampicin to 10 months for children with miliary tuberculosis. We treated children with a diagnosis of multidrug-resistant tuberculosis with second-line agents on the basis of antibiotic susceptibility results as previously described[17].

***Tuberculin skin test***

TST was administered to all children by the Mantoux method using 0·1 mL (2 tuberculin units) of purified protein derivative (PPD) RT23 (Statens Serum Institut, Copenhagen, Denmark). The test was performed and read by the study pediatrician who was blinded to ELISpot results. The cutaneous appearance of peau d’orange was noted in all participants, confirming intradermal inoculation of PPD. Induration was measured after 72 to 96 hours with a ruler. Induration was measured in millimetres across the forearm, perpendicular to the long axis, in accordance with the Centers for Disease Control and Prevention (CDC) guidelines[51].

***ELISpot Assays***

A 10 mL venous blood sample was taken for the ELISpot assay performed as previously described.[30,38] Briefly, pre-coated interferon-γ (IFN−γ) ELISpot plates (Mabtech AB, Stockholm, Sweden) were seeded with 2·5 x 10^5^ peripheral blood mononuclear cells per well: duplicate wells contained no antigen (negative control), phytohemagglutinin (positive control; ICN Biomedical, OH, USA) at 5 μg/mL, streptokinase-streptodornase (non-*M. tuberculosis* related antigen) at 20.8 IU/mL and a further 15 pairs of duplicate wells, each containing purified protein derivative (PPD) at 16.7 μg/mL and 1 of 14 peptide pools which incorporated 5, 6 or 7 15mer peptides from 96 such peptides spanning the length of ESAT-6 or CFP10, or selected regions[30] from Rv3873, Rv3878 and Rv3879c such that the final concentration of each peptide was 10 μg/mL. Previously described non-specific peptides from Rv3873 were excluded[30]. After overnight incubation at 37°C in 5% CO_2_, the plates were developed with preconjugated detector antibody (Mabtech AB) followed by chromogenic substrate (Moss Inc., Pasadena, MD, USA).

ELISpot plates were scored in Oxford by an automated ELISpot counter (AID-GmbH, Strassberg, Germany). Intensity and spot size settings were pre-defined and the same settings were used throughout. Mean readings from duplicate wells were electronically transferred to a spreadsheet by a customised software programme, ELISTAT (AID-GmbH, Strassberg, Germany). Responses were scored as positive if the test wells contained a mean of at least 5 spot-forming cells more than the mean of the negative control wells, and, in addition, this number was at least twice the mean of the negative control wells. Persons performing and reading the assays were blind to all personal identifiers and TST results.

*Ex-vivo* IFN-γ ELISpot assays were repeated 6 months post recruitment, including the negative and positive controls as described above. However, the repeat assays only included a further 6 pairs of duplicate wells, each containing 1 of 6 pools which incorporated 5 or 6 15mer peptides from 35 such peptides spanning the length of ESAT-6 or CFP10.
